# Short-Term Follow-Up Study on the Efficacy and Safety of Intravitreal Bevacizumab for Macular Edema Secondary to Retinal Venous Occlusions

**DOI:** 10.7759/cureus.88773

**Published:** 2025-07-25

**Authors:** Ramya Kandukuri, Sativada Lakshmi, Vudayana Dinesh Kanth

**Affiliations:** 1 Ophthalmology, Great Eastern Medical School and Hospital, Srikakulam, IND

**Keywords:** branch retinal vein occlusion, central retinal vein occlusion (crvo), diabetes, hypertension, intravitreal bevacizumab, macular edema, smoking

## Abstract

Aim

This study aimed to assess the short-term safety and efficacy of intravitreal bevacizumab in the treatment of macular edema (ME) secondary to retinal vein occlusion (RVO).

Methods

A prospective interventional study was conducted from July to December 2023, involving 30 patients with ME due to RVO. Intravitreal bevacizumab (1.25 mg/0.05 mL) was administered every 4-6 weeks. Follow-up evaluations included comprehensive ophthalmic examinations and central retinal thickness (CRT) measurements using optical coherence tomography, performed at baseline and throughout the six-month follow-up.

Results

The study included 30 patients with a mean age of 55.3 ± 10.21 years. Baseline CRT was 568.53 ± 156.29 µm, which significantly reduced to 309.53 ± 89.11 µm after six months. Best-corrected visual acuity (BCVA) improved from 1.14 ± 0.38 logMAR at baseline to 0.53 ± 0.19 logMAR at six months (p < 0.001). BCVA improvement was observed in 86.5% of patients. A gain of more than three lines was noted in 36.6% (n = 11), two or more lines in 46.6% (n = 14), and one line in 3.33% (n = 1). On average, patients received three injections (range: 1-3). No improvement was seen in 10% (n = 3), while deterioration occurred in 3.33% (n = 1).

Conclusion

Intravitreal bevacizumab appears to be a safe and effective short-term treatment for reducing ME and improving visual acuity in patients with RVO, based on outcomes observed at a tertiary referral eye hospital.

## Introduction

Retinal vein occlusion (RVO) affects an estimated 16.4 million people globally and is considered the second most common retinal vascular disorder after diabetic retinopathy. It is a significant cause of irreversible vision loss across various populations [1]. RVO is broadly categorized into three clinical types based on the site of vascular blockage and the extent of retinal involvement: central retinal vein occlusion (CRVO), branch retinal vein occlusion (BRVO), and hemicentral retinal vein occlusion (HCRVO).

Among these, BRVO is notably more prevalent, occurring four to six times more frequently than CRVO [2].

The condition predominantly affects middle-aged and elderly individuals and is often associated with systemic conditions that disrupt vascular integrity, such as chronic hypertension, arteriosclerosis, dyslipidemia, type 2 diabetes mellitus, thrombotic disorders, cerebrovascular disease, and elevated plasma viscosity [3]. Metabolic syndrome has also been recognized as a broader risk model for RVO. A male predominance has been reported in several studies. Additionally, smoking, due to its vasoconstrictive and pro-thrombotic effects, is strongly implicated as a modifiable risk factor for all RVO subtypes. Despite their anatomical differences, CRVO, BRVO, and HCRVO share similar systemic risk factors and microvascular pathologies [4].

One of the most common and visually debilitating complications of RVO is cystoid macular edema (ME), which leads to substantial central vision loss [5]. In recent years, the management of RVO-associated ME has shifted toward anti-vascular endothelial growth factor (anti-VEGF) agents, which inhibit VEGF, a key protein in angiogenesis and increased vascular permeability [6]. Bevacizumab, a recombinant humanized monoclonal antibody targeting VEGF-A, has shown promise in reducing ME by stabilizing the retinal vasculature and preventing abnormal vessel growth and leakage [7]. Compared to corticosteroid-based intravitreal therapies, which can also reduce ME, bevacizumab is less likely to cause complications such as elevated intraocular pressure and cataract formation [8].

The pathogenesis of ME and neovascularization in RVO is primarily driven by retinal hypoxia, which upregulates VEGF expression. This leads to increased vascular permeability and fluid accumulation, resulting in progressive visual deterioration [9]. Targeted inhibition of VEGF has become a central strategy in treating RVO, with multiple studies confirming its benefit in both visual and anatomical outcomes [10].

This study aims to evaluate the short-term safety and therapeutic response of intravitreal bevacizumab in patients with ME secondary to RVO. By assessing changes in central retinal thickness (CRT) and visual acuity, along with any adverse effects, this study hopes to add to the growing clinical evidence supporting the use of anti-VEGF therapy in retinal vascular diseases.

## Materials and methods

This non-randomized, open-label, prospective interventional study was conducted in the Department of Ophthalmology at Great Eastern Medical School and Hospital, Ragolu, Andhra Pradesh. The study period spanned from July to December 2023 and was approved by the Institutional Ethics Committee (IEC) under approval reference number 105/IEC/GEMS&H/2023, adhering to ethical guidelines for research involving human participants.

A total of 30 patients who consecutively presented to the ophthalmology outpatient department and exhibited clinical signs of RVO (either CRVO or BRVO) were enrolled. Diagnosis was confirmed through detailed fundus examination and visual acuity testing. Eligibility criteria included the presence of ME, defined as a CRT greater than 249 microns, and a BCVA below 6/12. All participants provided written informed consent and demonstrated a willingness and ability to comply with follow-up visits over the six-month observation period.

Inclusion criteria

Participants eligible for recruitment included individuals diagnosed with retinal vein occlusion syndrome (RVOS) who presented with clinically confirmed ME and associated visual impairment. Only those who provided documented informed consent after a thorough clinical explanation of the study were enrolled.

Exclusion criteria

Systemic

Subjects were excluded if they had poorly controlled systemic hypertension or diabetes mellitus, a history of thromboembolic events, ongoing anticoagulant therapy, or were pregnant.

Ocular

Individuals were deemed ineligible if they had pre-existing retinal disorders such as diabetic retinopathy, age-related macular degeneration, macular scarring, or other vision-affecting ocular pathologies, including significant media opacities, history of uveitis, epiretinal membrane (ERM) involving the macula, or other optic neuropathies. Additional exclusion criteria included prior treatment with intravitreal anti-VEGF or corticosteroids, previous retinal laser procedures, neovascular glaucoma, fundus obscuration, or RVO secondary to elevated intraocular pressure.

All consenting participants underwent a detailed clinical interview, including demographic data, symptom duration, presenting ocular complaints, and associated systemic illnesses such as hypertension, diabetes, cardiovascular disease, and lipid disorders.

A comprehensive ophthalmic evaluation was conducted, consisting of uncorrected and best-corrected visual acuity (BCVA) testing using the Snellen chart, followed by slit-lamp biomicroscopy and posterior segment examination with a 90D lens and indirect ophthalmoscopy. Retinal pathology was documented via color fundus photography.

CRT was measured using high-resolution spectral-domain optical coherence tomography (SD-OCT) [11], performed at baseline and at 4-6-week intervals thereafter. Intraocular pressure (IOP) was assessed using Goldmann applanation tonometry. Systemic blood pressure was measured at each visit, and all patients were advised to undergo fasting and postprandial blood glucose testing along with a lipid profile to assess vascular risk. Where indicated, patients were referred to internists or cardiologists for systemic optimization before initiating ocular treatment.

Intravitreal bevacizumab (1.25 mg in 0.05 mL) was administered via the pars plana using a 27- or 30-gauge needle under sterile conditions in a dedicated procedure suite. The first injection was given at enrollment, with subsequent doses determined by disease activity observed on follow-up. Post-injection, patients were prescribed topical gatifloxacin 0.3% eye drops four times daily for one week to minimize the risk of endophthalmitis.

At each follow-up visit, patients underwent BCVA reassessment, anterior and posterior segment evaluation, and SD-OCT imaging to monitor ME. Recurrence of ME was defined as any increase in CRT from the previous visit. Retreatment was indicated if CRT increased by ≥100 µm, with or without a reduction in visual acuity of at least one Snellen line (five ETDRS letters).

Visual acuity readings were converted into logMAR units for standardized analysis. Statistical evaluations were conducted using SPSS software, version 13.0 (SPSS Inc., Chicago, IL, USA). Paired t-tests were used to compare baseline and post-treatment outcomes, with a p-value < 0.05 considered statistically significant.

## Results

This study included 30 eyes from 30 individual patients. The mean age of the cohort was 55.3 ± 11.67 years, with participants ranging from 40 to 77 years old. Male patients comprised 53.3% (n = 16) of the study population, while females accounted for 46.7% (n = 14), as detailed in Table 1. The average duration of symptoms prior to presentation was 1.0 ± 0.48 months, with onset ranging between two weeks and two months.

**Table 1:** Demographic and clinical characteristics BRVO: branch retinal vein occlusion, CRVO: central retinal vein occlusion.

Category	Sub-category	No. of cases (n)	Percentage (%)
Sex distribution	Males	16	53.30%
	Females	14	46.70%
	Total	30	100%
Type of occlusion	BRVO	17	56.70%
	CRVO	13	43.30%
	Total	30	100%
Associated factors	Hypertension	13	43.30%
	Diabetes mellitus	6	20.00%
	Hypertension + diabetes	4	13.30%
	Smoking	7	23.30%
	Total	30	100%

In terms of RVO subtype distribution, 56.7% (n = 17) were diagnosed with BRVO, and 43.3% (n = 13) had CRVO. The mean number of intravitreal bevacizumab injections per patient was 3 ± 0.96, with individual patients receiving between one and four injections (Table 2).

**Table 2:** General characteristics of patients

	Minimum	Maximum	Mean	SD
Age (years)	40	77	55.3	10.21
Number of intravitreal bevacizumab injections	1	4	3	0.96

Assessment of systemic comorbidities revealed arterial hypertension as the most prevalent condition, present in 43.3% (n = 13) of participants. Type 2 diabetes mellitus was observed in 20% (n = 6), and 23.3% (n = 7) reported a history of tobacco use. Concurrent diagnoses of both hypertension and diabetes were noted in 13.3% (n = 4) of the cohort.

Follow-up outcomes

Visual Acuity

BCVA, initially measured using the Snellen chart and converted to logMAR units for standardized analysis, demonstrated significant improvement over the course of treatment. At baseline, the mean BCVA was 1.14 ± 0.38 logMAR. This improved to 0.82 ± 0.29 at six weeks, 0.63 ± 0.20 at three months, and 0.53 ± 0.19 at the six-month follow-up. All improvements from baseline were statistically significant (p < 0.0001), as summarized in Table 3.

**Table 3:** Best-corrected visual acuity after intravitreal bevacizumab p-values represent comparison with the baseline values using a paired t-test.

BCVA	Mean (in logMAR units) ± SD	p-value
Baseline	1.14 ± 0.38	–
At six weeks	0.82 ± 0.29	<0.0001
At three months	0.63 ± 0.20	<0.0001
At six months	0.53 ± 0.19	<0.0001

At the six-month follow-up, 86.5% (n = 26) of participants exhibited an overall improvement in BCVA. A gain of more than three lines was noted in 36.6% (n = 11), two or more lines in 46.6% (n = 14), and a one-line improvement in 3.33% (n = 1). Visual acuity remained stable in 10% (n = 3) of cases, while a slight decline was observed in 3.33% (n = 1).

Central Retinal Thickness

Serial OCT measurements demonstrated a significant reduction in central retinal thickness (CRT) throughout the study. The mean baseline CRT was 568.53 ± 156.29 μm, which decreased to 403.03 ± 86.94 μm at six weeks, 344.46 ± 95.94 μm at three months, and 309.53 ± 89.11 μm at six months. These reductions were statistically significant at all time points (p < 0.0001), as detailed in Table 4.

**Table 4:** Status of central retinal thickness (CRT) after intravitreal bevacizumab p-values represent comparison with the baseline values using a paired t-test.

	Mean CRT (μm)	p-value
Baseline	568.53 ± 156.29	–
Six weeks	403.03 ± 86.94	<0.0001
Three months	344.46 ± 95.94	<0.0001
Six months	309.53 ± 89.11	<0.0001

Safety

Throughout the six-month treatment period, no major ocular or systemic adverse events were observed. Notably, there were no cases of IOP elevation, retinal detachment, endophthalmitis, or thromboembolic complications. The absence of treatment-related side effects highlights the favorable short-term safety profile of intravitreal bevacizumab in this patient cohort.

## Discussion

This prospective interventional study evaluated the short-term outcomes of intravitreal bevacizumab, an anti-VEGF agent, administered every 4-6 weeks in patients with ME secondary to RVO. Over a six-month follow-up period, the treatment demonstrated significant improvement in both central macular thickness (CMT) and BCVA, affirming its efficacy and safety.

A total of 30 eyes with RVO-related ME were treated with bevacizumab. The intervention was well tolerated, and no serious ocular or systemic adverse effects were observed, supporting its favorable safety profile. The mean age at presentation was 55.3 years, closely paralleling findings from Thakur et al. [12], who reported mean ages of 58.54 and 56.6 ± 11.51 years. A slight male predominance (53%) was noted, consistent with data from Kumar et al. [13].

In terms of RVO subtypes, BRVO accounted for 57% of cases, while CRVO comprised 43%, mirroring the distribution trends noted by Bhandari et al. [14]. Systemic comorbidities were prevalent, with hypertension present in 43.3% of participants, diabetes in 20%, a history of smoking in 23.3%, and 13.3% exhibiting both hypertension and diabetes.

Our results are in agreement with those of Stahl et al. [15], who emphasized that early administration of bevacizumab (within three months of CRVO onset) can lead to better visual outcomes. The mean number of injections per eye was 3 ± 0.96, similar to the frequency reported by Delsoz et al. [16]. BCVA improved significantly over time, from a mean of 1.14 logMAR at baseline to 0.53 logMAR at six months (p < 0.001), with 86.5% of eyes showing visual improvement. Gains of more than three lines were recorded in 36.6% of cases, over two lines in 46.6%, and one-line improvement in 3.3%. These trends closely match the outcomes reported by Thakur et al. [12].

Gender distribution in our cohort aligns with observations by Hamam et al. [17], while the prevalence of hypertension as a systemic risk factor is corroborated by Thakur et al. [12] and Parajuli et al. [18].

Anatomical outcomes also supported treatment efficacy. Mean CMT decreased from 568.53 ± 156.29 µm at baseline to 309.53 ± 89.11 µm at six months (p < 0.0001). These results are consistent with reductions reported by Thakur et al. [12], Parajuli et al. [18], Hamam et al. [17], and Bhandari et al. [14].

Additionally, findings are supported by Hall et al. [19], whose randomized controlled trial showed an average gain of 14.1 ETDRS letters in the treatment group compared to a 2.0-letter loss in controls, reinforcing the visual benefits of bevacizumab. No significant changes in IOP were observed in either BRVO or CRVO groups during the follow-up period, further affirming the treatment’s ocular safety.

Limitations

This study is limited by its relatively small sample size and short follow-up duration, which may limit the generalizability of findings. Furthermore, inconsistent control of systemic conditions such as hypertension and diabetes, likely due to limited patient awareness, may have influenced treatment outcomes, making it challenging to isolate the effect of anti-VEGF therapy alone. As such, conclusions regarding optimal treatment frequency and long-term efficacy should be interpreted with caution.

## Conclusions

This study highlights the clinical utility of intravitreal anti-VEGF therapy, specifically bevacizumab, in the management of ME secondary to RVOs. The treatment produced consistent and substantial reductions in central macular thickness, along with meaningful improvements in visual acuity, sustained over a six-month follow-up period. Most patients required no more than three injections to achieve these anatomical and functional benefits. The absence of significant ocular or systemic complications further supports the favorable safety profile of bevacizumab. Additionally, the findings underscore the influence of systemic factors such as hypertension, diabetes, and smoking in the pathogenesis of RVO, emphasizing the importance of comprehensive, multidisciplinary patient care. While the results are encouraging, future studies with larger sample sizes and longer follow-up durations are warranted to refine treatment protocols and confirm long-term outcomes.
